# The “home-based exercise for breast and prostate cancer patients during treatment—a feasibility trial” (BENEFIT CA trial): rationale and methodological protocol

**DOI:** 10.1186/s40814-023-01393-0

**Published:** 2023-09-26

**Authors:** Larissa Xavier Neves da Silva, Jayne Santos Leite, Andresa Conrado Ignacio, Fernanda Dias Massierer, Lucinéia Orsolin Pfeifer, Linda Ariene dos Santos Cardoso, Tainá Silveira Alano, Daniel Umpierre

**Affiliations:** 1 LADD Lab, Hospital de Clínicas de Porto Alegre, Centro de Pesquisa Clínica, Porto Alegre, RS Brazil; 2 Postgraduate Program in Health Sciences (Cardiology and Cardiovascular Sciences), Universidade Federal Do Rio Grande Do Sul, Porto Alegre, RS Brazil; 3 Biomedicine School, Universidade Federal de Ciências da Saúde de Porto Alegre (UFCSPA), Porto Alegre, RS Brazil; 4 Medical School, Universidade Federal de Ciências da Saúde de Porto Alegre (UFCSPA), Porto Alegre, RS Brazil; 5 Department of Public Health, Universidade Federal do Rio Grande do Sul (UFRGS), Porto Alegre, RS Brazil; 6 National Institute of Science and Technology for Health Technology Assessment (IATS/HCPA), Hospital de Clínicas de Porto Alegre, Centro de Pesquisa Clínica, Porto Alegre, RS Brazil

**Keywords:** Feasibility, Home-based, Physical activity, Exercise, Cancer

## Abstract

**Background:**

Physical activity has been shown to benefit patients undergoing adjuvant cancer therapy. Although exercise interventions may be applied in several settings, most trials have focused on specialized facilities for their interventions. While these approaches benefit the access for individuals living near exercise centers, it hampers the assessment of real-world effectiveness. Therefore, evaluating the feasibility and implementation of home-based models of exercise training, especially in low-to-middle-income settings, may inform future physical activity trials and programs. In this article, we present the protocol for the BENEFIT CA trial, which aims to assess the implementation of a remote exercise intervention for patients with breast cancer or prostate cancer, primarily quantifying adherence to an exercise program.

**Methods:**

This is a 12-week study, utilizing a non-randomized, single-arm design to assess the feasibility of a home-based exercise training. The intervention is remotely guided, and participants also receive an educational component about cancer and exercise. The study aims to recruit 40 patients diagnosed with breast cancer and 40 patients diagnosed with prostate cancer, all of whom undergoing active hormonal treatment. The primary outcome is the level of adherence, indicated as the proportion of performed exercise episodes. Secondary outcomes include recruitment rates, fatigue, quality of life, and functional capacity. Adverse events will be monitored throughout the study. Because this is a feasibility trial, the statistical analysis plan is based on descriptive statistics, which encompasses an intention-to-treat analysis and a plan for handling missing data.

**Discussion:**

This is a low-cost feasibility study to orient the design of a wide-range, pragmatic phase 3 trial based on remote exercise intervention. With this study, we aim to better understand the adherence and implementation strategies regarding home-based exercise for the proposed population and, in the near future, move forward to a randomized clinical trial. In addition, this trial may contribute to engage patients with cancer in exercise programs throughout their treatment and beyond.

**Trial registration:**

This trial has been approved by the Hospital de Clínicas de Porto Alegre Ethics Committee/IRB (48,869,621.9.0000.5327), and it is registered at Clinicaltrials.gov (NCT05258526), registered on February 25, 2022, prior to the beginning of the study.

## Background

There is a growing body of evidence indicating physical exercise plays a protective role against several types of cancer [1, 2], with a recent development of a Brazilian physical activity guide regarding recommendations to prevent and manage cancer [3]. Moreover, beyond the association with reduced risk of different types of cancer, exercise has been indicated during and after cancer treatment to improve overall fitness as well as to counterbalance side effects from pharmacological and surgical treatments [4, 5]. In addition, emerging studies have been exploring the potential association between physical activity and a reduced risk of breast cancer recurrence [4–6], further highlighting the potential benefits of an active lifestyle for individuals with cancer.

Numerous studies have investigated the relationship between cancer and exercise [1, 2, 6, 7], examining its impact before, during, and after treatment. Notably, the majority of these trials have been conducted in high-income countries, using supervised, face-to-face interventions [8–10]. However, many face-to-face programs rarely translate into public-wide implementation, since they are not designed to amplify the exercise strategies outside the training facilities [11–13]. Other difficulties to attend exercise sessions are associated with economic status, treatment side effects, and aesthetic factors that may influence the engagement in a face-to-face exercise program [4, 14]. To address this issue, other formats for exercise could be investigated as alternatives to amplify physical activity (PA) opportunities for the cancer population undergoing active treatment. With a remote follow-up, patients with cancer may achieve good levels of physical activity in an easier way and, even if the conditions are not ideal, enjoy the benefits of exercise training. The COVID-19 pandemic has prompted a rapid upsurge in the application of technology, providing a safe and affordable means of contact for patients and researchers. Nevertheless, the urgency to promote online care using smartphones and other remote ways should not reduce the attention to the promotion of patient engagement. With this in mind, it is important to analyze if these models are well accepted and feasible for patients with cancer [15].

Therefore, this study addresses the amplification of PA for individuals with cancer undergoing active treatment for breast or prostate cancer. In this regard, we seek to test a low-cost, pragmatic intervention designed to increase the number of patients involved in exercise programs. The study consists of a feasibility trial for the reduction in methodological uncertainties regarding the implementation of the home-based strategy, which will be potentially tested in a future phase-three randomized trial. This study intends to characterize the participant’s level of adherence, as well as deepen the knowledge of possible barriers and facilitators for this type of intervention.

The main objective of this study is to evaluate the feasibility of a home-based exercise combined training by measuring adherence rates. The intervention includes educational contents related to health and cancer, which will be delivered through telephone, text messages, or email. The secondary objectives are to (1) evaluate the quality of life and fatigue levels, as well as their magnitude variations, through validated questionnaires, before and after a 12-week home-based combined training, with a remotely guided follow-up, and (2) assess the effects of 12-week home-based combined training on functional capacity, measured by 6-min walk test and hand grip strength. This study, being a feasibility trial, does not entail a formal hypothesis to be tested.

## Methods

This single-arm feasibility trial was approved by Hospital de Clínicas de Porto Alegre (Ethics Committee/IRB: 48869621900005327) in the year of 2022. The trial will substantiate the decision regarding the design of a phase-three randomized clinical trial with remote approaches and clinical outcomes for cancer. This study, therefore, focuses on trial recruitment and patient acceptability, considering the disease and treatment aspects that may influence adherence to the proposed intervention. To qualify a possible future trial, this study might be used to facilitate public and patient involvement [16, 17].

### Sample size calculation

Because this is a feasibility study, the study may preclude formal sample size calculation. Therefore, we predefined a total sample target of 80 participants, resulting in 40 patients for each cancer type (breast and prostate cancer). Assuming a potential dropout rate of 50%, we could anticipate that at least 20 participants for each cancer would complete the study.

### Outcomes

The study’s primary outcome is the participants’ level of adherence to the planned exercise episodes. This outcome is measured by the cumulative number of times participants self-report engaging in the exercise program from a total of 36 sessions prescribed in the program. This outcome is measured through weekly assessments carried out by phone call or text message. The cumulative number of self-reported engagement in sessions will be converted to percentages.

This study also addresses variables that may be relevant to methodological feasibility, participant safety, and potential direct effects of the exercise program, as follows:

1. Participate recruitment and engagement: this includes the number of participants selected and consenting to participate in the study, how participants were recruited, and participant attrition. This variable is assessed from the first contact with a potential individual with breast or prostate cancer until a decision regarding eligibility and study participation is reached [18].
2. Adverse events: the study team conducts weekly checks by phone call with participants to identify any potential adverse events, such as physical injury or mental/emotional issues.
3. Fatigue and quality of life: these variables are assessed using the Functional Assessment of Cancer Therapy Questionnaire [19]. The questionnaire comprises specific items that capture various aspects of fatigue and quality of life, and these items are combined to generate standardized values. The questionnaire is used in a pre-/post-text format in order to identify change throughout the study.
4. Functional capacity: a pre-/post-format is used to determine any changes in participants’ functional capacity. These tests are conducted in person to measure participants’ performance on the 6-min walk test and the hand-grip test.

### Open feedback from participants

At the conclusion of the study, participants will be invited to participate in a focus group, wherein discussion questions pertaining to the study’s intervention and outcomes will be used. The mode of conducting the focus group, either in-person or virtually, will be determined based on the availability and preferences of the participants.

### Additional measures

To analyze the participants’ initial PA levels, we will utilize the International Physical Activity Questionnaire (IPAQ), which will be administered at the beginning of the study. In addition, height, body weight, and abdominal circumference will be measured in-person, in a pre-/post-format. Lymphedema control circumferences will be measured in a pre-/post-format for female participants, regardless of their history of lymphedema. This measurement aims to provide participants with information about the limits of PA following surgery.

### Study procedures

**Fig. 1:**
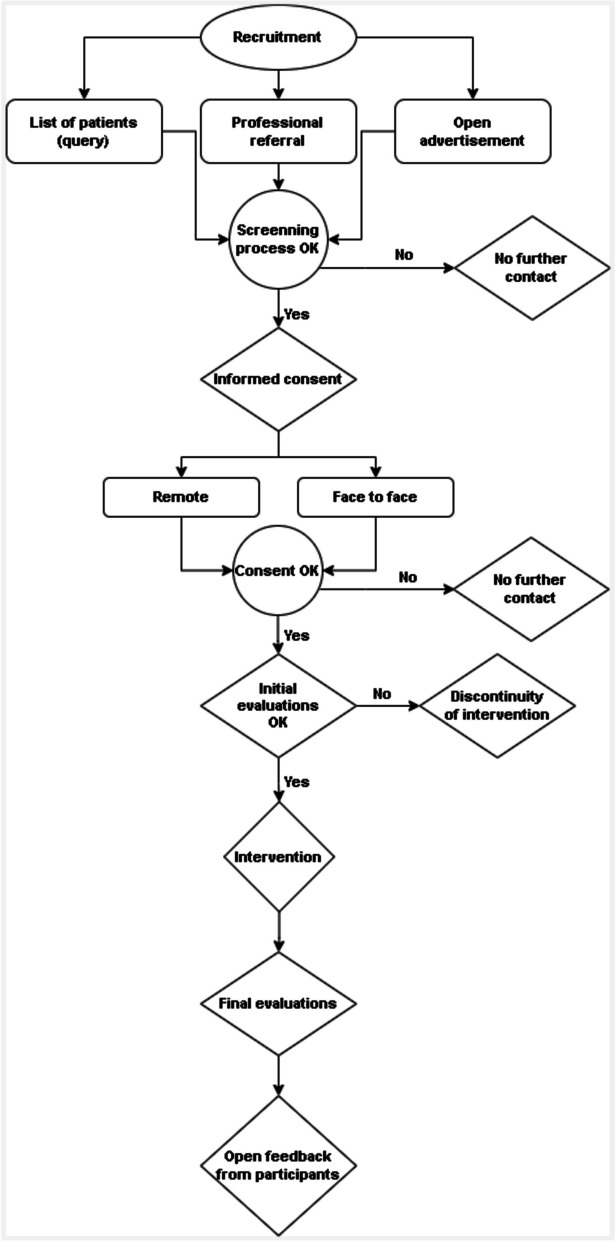
Study flow diagram

**Fig. 2:**
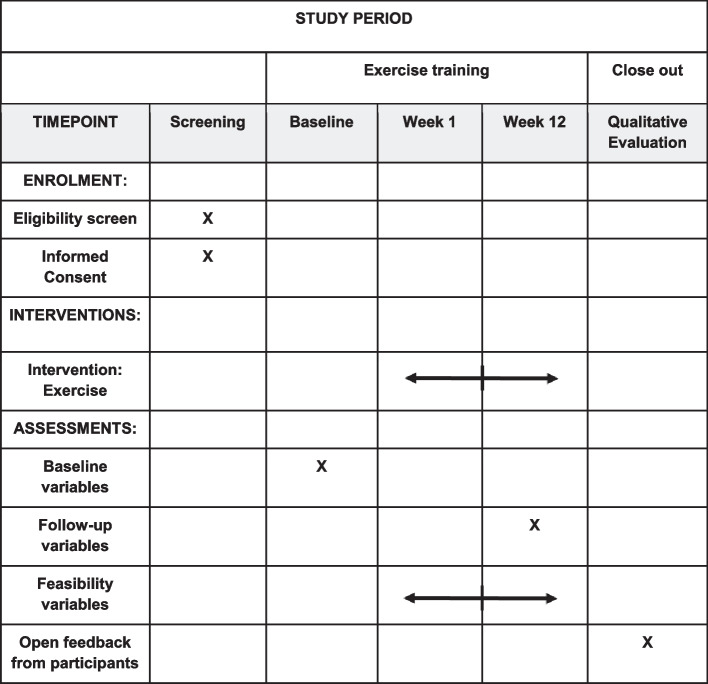
Study timetable with a specific period. SPIRIT figure regarding the study’s phases

This study accounts for eligibility screening and recruitment, participant consent, initial evaluations, intervention, final evaluations, and hearing for open feedback, as stated in the figure below (Fig. 1). Moreover, we have developed this protocol in accordance with the Standard Protocol Items: Recommendations for Interventional Trials (SPIRIT) guidelines [20] and have incorporated the CONSORT 2010 statement: extension to randomized pilot and feasibility trials [21], whenever applicable. Figure 2 shows the SPIRIT timetable.

### Eligibility screening and recruitment

To assess the eligibility of prospective individuals, a comprehensive screening plan was developed and is publicly available at the following link: (https://osf.io/n7g5p). The plan is detailed and thoroughly explained. This screening plan is uniformly employed for all potential participants, regardless of how they are recruited to the study. The study uses three methods of recruitment, which are as follows:

1. Referral from the hospital lists: this method involves obtaining patient lists from the oncology and urology clinics at the Hospital de Clínicas de Porto Alegre, Brazil. The lists include patients currently receiving treatment at the hospital.
2. Referral from professionals: In this approach, patients who appear to meet the eligibility criteria for the study are referred by healthcare professionals, such as oncologists, who have treated patients in their offices. These professionals identify patients who could potentially participate in the study and refer them to the study recruiting team.
3. Open advertisement: use of media, such as newspapers and social media (Instagram and Whatsapp) for propagation of the study.

Nevertheless, because of recruitment rates from the initial weeks of the project, we already have put to use the open advertisement.

### Inclusion criteria

The following are the breast cancer patients’ inclusion criteria:

- Age equal or superior to 18 years old.
- Living in the city of Porto Alegre or Porto Alegre Metropolitan Region.
- Diagnosis of breast cancer stages 0 to III.
- Undergoing hormonal treatment/manipulation isolated or combined with other methods.
- Initiation of hormonal treatment/manipulation (such as anti-estrogen therapy) isolated or combined to others within the 3 months before enrolment and continued treatment at the beginning of the trial.
- Planning for consistent use of the hormone manipulation treatment throughout the intervention period.
- Ability to independently perform PA, including walking without assistance and engaging in daily chores such as standing up and sitting down a chair. This capability is verified during the initial contact by phone and in-person during the initial assessments.
- Not regularly practicing physical exercise (more than once a week) for the past 3 months.
- In case the patient has undergone some surgical procedure, they must have medical clearance for exercise training before participating in the study.

The following are the prostate cancer patients’ inclusion criteria:

- Age equal or superior to 18 years old.
- Living in Porto Alegre or Porto Alegre Metropolitan Region.
- Localized prostate cancer diagnosis.
- Having initiated the hormonal treatment/manipulation (such as anti-androgen therapy) isolated or combined to others in the 3 months prior to the study enrollment and being under this treatment at the beginning of the trial.
- Ensure consistent and planned administration of hormonal therapy/manipulation throughout the entire intervention period.
- Ability to perform PA on their own, such as being able to walk without assistance and perform daily chores, such as standing up and sitting down a chair. This is verified during the initial contact by phone and in-person, during the initial evaluations.
- Not regularly practicing physical exercise (more than once a week) for the past 3 months.
- In case the patient has undergone some surgical procedure, they must have medical clearance for exercise training before participating in the study.

### Exclusion criteria

The following are the exclusion criteria:

- Patients presenting metastatic disease or active loco-regional prior to their enrollment.
- Inability to understand the terms and study conditions, language, hearing and cognition difficulties, or any major psychiatric issues or hindrances.
- Simultaneous participation or a family member in the same household, already engaged in the study.
- Moving plans or a trip that causes an absence greater than 2 weeks throughout the study length.
- Medical history of cardiovascular disease (with the exception of hypertension under the use of medication) or severe cardiopulmonary disease, such as history of heart attack, revascularization procedures, deep venous thrombosis, cerebrovascular accident, or pulmonary embolism in the past 12 months.
- Chronic pulmonary disease requiring use of oxygen or corticosteroid therapy.
- Kidney disease with use or about to initiate dialysis, or yet, on the waitlist for a kidney transplant.
- Severe nausea, anorexia, or any other condition that does not allow the performance of exercise;
- Presentation of a medical report stating any contraindications or medical conditions that are incompatible with exercise training.

### Patient consent

The patient consent is set by the reading, clarifications, and agreement of the informed consent form (https://osf.io/f24dn). This study utilizes two consent form procedures: a remote form, sent by email for the patient to read and decide his/her consent to participate in the study, and a printed form, which the patient signs during the face-to-face meeting, as requested by the local IRB. In addition, the consent form has two consenting parts: one related to consent to participate in the study and another related to data sharing in an anonymized approach, in a public repository. For example, if the patient, by any reason, cannot go to the hospital facilities to take part in the evaluations, the research team can visit the patient at his/her home.

### Initial evaluations

#### Health, demographic, fatigue, quality of life, and physical activity questionnaires

All the questionnaires are answered (after the completion of the informed consent form). To note, sanitary recommendations set by the WHO [22] have been followed, in case of in-person contact.

The measurements of fatigue and quality of life scores are achieved through answering of specific scales for each sex: women will answer the *Functional Assessment Cancer Therapy Breas*t (FACT-B) questionnaire, on its Portuguese version, whereas men will answer the *Functional Assessment Cancer Therapy Prostate* (FACT-P) questionnaire, on its Portuguese version. Such questionnaires are derived from the questionnaire *Functional Assessment of Cancer Therapy—General* (FACT-G), which is also being applied to the participants. The FACT-B and FACT-P comprise the instrument FACT-G and the domain “Additional Worries,” composed by 10 and 12 questions regarding specifically to symptoms and issues from breast and prostate cancer, respectively. In total, we have 37 and 39 questions divided into 5 domains (physical well-being, social/family, emotionally, functionally, and additional worries). Each answer may vary from 0 (worst state of health) to 4 (best state of health).

PA is measured through the IPAQ, in its long version, in Portuguese. This instrument has 5 domains (PA at work, PA as a means of transportation, home chores, recreational activities, sport, leisure, and sitting time), totalling 27 items.

#### Six-minute walk test (6 MW)

The 6-min walk test 6 MW assesses an individual functional capacity to continuous walking for 6 min at a steady pace, aiming to cover the highest distance in meters. This procedure is held in a flat environment, 25 m wide, with visual markers placed every 3 m. The subjective effort is measured by the Borg scale at the beginning and at the end of the test. Standard stimuli are given to each individual at every minute of the test [23].

#### Handgrip test

The handgrip strength is assessed by manual dynamometry in both hands. The test consists of 3 attempts in each hand, with a minute apart from each attempt. The individual stands, with elbows flexed in a 90° angle and performs the maximum possible strength for 4 s. The highest value is chosen as the result of the test [24].

#### Anthropometric profile

To characterize the anthropometric profile, body mass (kg), height (cm), and abdominal circumference (cm) are measured, following the standards from the International Society for Advances in Kinanthropometry (ISAK). Besides this, there are measures of lymphedema control for patients with breast cancer, with measurements at four circumference points: metacarpophalangeal joint, fist, 10 cm away from the lateral epicondyle and 12 cm in proximity to the lateral epicondyle regarding the superior limbs from each female patient, in a pre-/post-format. Differences larger than 2 cm at any point represent a statistical difference and, therefore, a lymphedema [25].

### Training simulation

The research team provides training simulations of the exercises when the participants visit the laboratory or receive at-home visits. In addition, participants receive virtual or printed material as a visual aid for their workouts. The research team records the technical aspects (e.g., movement specificities) regarding the exercise performance so that these may be approached, if needed, in the follow-up telephone calls.

### Intervention, contacts, and adverse events

The trial intervention was designed taking into account three major aims: (1) to offer a pragmatic intervention that could be adaptable for the public health system and scaled up depending upon the available setting for exercise training, (2) to implement an exercise program jointly to an educational component, and (3) to implement the intervention as remotely as possible so that the intervention could reduce accessibility and mobility barriers to exercise.

Both breast and prostate cancer patients receive, prior to the beginning of the trial, the same instructions to exercise at their homes, parks, or other places they feel comfortable, three times a week, at hours and days of their choice. Telephone calls, text messaging, and e-mail are conducted to follow up the patients’ progression and their health status, to record possible issues, and to motivate them to continue exercising (all material is available at https://osf.io/3zcfn/).

During the weekly contact with the participant, adverse events that may have occurred are accounted for. Events or issues throughout the study length (e.g., sickness, fall, neuromuscular injury) are computed as adverse events, being classified according to their severity (mild, moderate, severe), predictability (expected or unexpected), and potential relation with study procedures (definitely related, possibly related, or unrelated).

### Final evaluations

Aside from the participant’s informed consent, the health and demographic questionnaires and the PA levels, fatigue and quality of life questionnaires, and anthropometric and functional capacity evaluations will be carried out again, during up to two visits to the laboratory or participants’ home.

### Statistical considerations

We will primarily follow intention-to-treat principles, therefore accounting for all participants in analyses, regardless of their level of adherence. One participant will be considered to be entered in analysis (analysis set) after completing baseline assessments. If the participant withdraws before the completion of baseline assessments, he/she will be presented in the flowchart but will be considered a loss before the intervention’s allocation.

Statistical data treatment will be mostly by descriptive statistics, through means or medians for discrete and continuous variables, and frequencies (absolute and relative) for categorical variables, with their respective precision measures (ranges or 95% confidence interval). The Shapiro–Wilk test will be carried out to assess whether the main continuous outcomes follow a normal distribution.

We will use general estimating equations (GEE) to compare means over time (pre-post-assessments). For categorical variables, we will use the McNemar test, comparing data regarding the pre- and post-intervention moments.

Regarding missing data, we will employ different strategies for different variables. For the quality of life and fatigue scores, the last observation carried forward (LOCF) imputation method will be used. For the training sessions, a missed value will be considered as a non-attendance, since the trial accounts for multiple forms of contact that can verify if the participant performed or not the session.

### Criterion to move forward to a phase 3 randomized controlled trial

The primary criterion for advancing to a randomized controlled trial (RCT) is based on the weekly adherence to the exercise sessions by at least 40% of our sample. To determine adherence, we defined a threshold of one or more exercise sessions per week for a minimum of 8 weeks, irrespective of the completion or non-completion of the intended exercise protocol. Consequently, if we have a total of 40 patients, and at least 16 of them consistently perform the exercise program more than once per week over 8 weeks, they will be classified as adherent, even if they did not fully complete the proposed exercises or if a session is partially incomplete.

In case the progression criterion is reached, other aspects such as adverse events, recruitment performance, overall satisfaction, and patient engagement will be considered to design a future trial.

### Ethical and safety issues

A brief guideline was developed for patients to observe themselves at the moment of engaging in exercise (material available at: https://osf.io/xjgfh). In addition, there are some safety criteria observed during the study’s achievement, such as:

- During the health questionnaire, the evaluations or the intervention, in case of any disorder, cardiopulmonary, and/or important neuromuscular dysfunction, there will be performed an additional analysis, along with the medical staff, to determine the continuity or not of the patient in the study.
- For any intercurrence that needs medical care, the researchers will immediately contact the emergency health centers from the surrounding regions.
- Absolute medical contraindications to patient participation in the study: recent modification in the rest electrocardiogram (ECG) indication of myocardial ischemia or other acute event (< 2 days), myocarditis or acute pericarditis, and systemic acute infection.
- Assessing medical eligibility and identifying contraindications for continued study participation: severe episode of hypertension (systolic blood pressure (SBP) > 200 mmHg or diastolic blood pressure (DBP) > 100 mmHg), tachydysrhythmia or bradyarrhythmia, hypertrophic cardiomyopathy, high degree atrium-ventricular blockage, decompensated metabolic disease, and chronic infectious disease.

The research team clarifies the participants that they can drop out the study at any time, for any reason they judge relevant. If a participant has a contraindication to exercise at a particular moment, this decision will be respected and the intervention will be discontinued. In addition, if there is any harm related to the intervention, or the occurrence of a major adverse event, the research team will provide assistance in a face-to-face or remote manner, contacting emergency and facilitating as much as possible the aspects related to the participant’s safety.

### Data management and dissemination

The handling, identification, and storage of generated and collected data, along with procedures regarding the data monitoring process, are described in our publicly available Data Management Plan (DMP; https://osf.io/8725a). According to the FAIR principles, this plan foresees the use of a standardized dictionary (NCI Thesaurus) for available variables, which seeks to increase tracking, interoperability, and possibility of research data. All the media materials used in this study have been made available for reuse throughout open platforms, such as Open Science Framework (OSF).

### Ancillary, post-trial care, and harm from trial participation

All participants will receive results from the assessment carried out during the study, with available information that may assist their cancer treatment and overall healthcare. However, due to the feasibility nature of the trial and non-pharmacological intervention, no formal post-trial care (e.g., continuation of exercise prescription) is planned to be provided. However, at the end of the feasibility trial, the patients will be called out to participate in a focus group to discuss their views and choices of outcomes of this and future trials.

## Discussion

This feasibility trial seeks to better understand and evaluate the home-based exercise training model, its implementation, and its acceptance by patients with breast or prostate cancer undergoing treatment. Since several clinical and contextual reasons may preclude patients from attending exercise training facilities, considering exercise at home could be beneficial and more inclusive for patients undergoing cancer treatment.

Although our intervention inevitably carries some risks, such as possible discomfort during the workouts, injuries related to exercise, excessive fatigue, muscle soreness, or others, we underscore that our aims include monitoring any adverse events. This contributes to characterizing potential harms induced by exercise training and tailor programs that may reduce or better handle such issues. Additionally, the use of web-based technology, through the use of phone messaging, may impair the intervention delivery for some participants. In this regard, we also offer conventional (phone or paper based) contacts and evaluations formats.

We anticipate that the motivation to exercise could be lower in remote intervention when compared with face-to-face approaches. Therefore, accounting for this potential scenario, the feasibility design allows us to “fail fast,” without the spending of major resources, especially reducing the burden for trial participants. Alternatively, in a best case scenario, in which we detect moderate to high adherence to the proposed intervention, we can proceed with necessary adjustments and expand this investigation to a confirmatory trial, with substantial public and participant involvement. Based on a flexible approach to contact patients, adequate contact frequency, and open channels for discussions and support, it is reasonable to expect this research program is well positioned to move forward.

In summary, this pragmatic, low-cost feasibility trial seeks to test a model to distribute exercise interventions more widely to people with breast or prostate cancer. Although exercise training has been consistently shown to benefit patients under active cancer treatment, reducing fatigue, improving neuromuscular status, and increasing quality of life [4, 5, 14, 26, 27], research oriented to a public health setting is needed to tailor interventions in a more equitable way, leaving no patients behind regarding their opportunities to engage in PA.

### Trial status

By the moment of the submission of the present protocol, 10 women and 3 men have been recruited and engaged in intervention.

### Registration

This trial has been approved by the Hospital de Clínicas de Porto Alegre Ethics Committee/IRB (48869621900005327), and it is registered at Clinicaltrials.gov (NCT05258526), registered on February 25, 2022, prior to the beginning of the study.

### Protocol version

This manuscript is in its first version, dated as of May 25th, 2023.

## Data Availability

Supplementary data and study materials are at the project’s OSF page, available at https://osf.io/3zcfn/.

## Abbreviations

CAPES: Coordenação de Aperfeiçoamento de Pessoal de Nível Superior
DMP: Data Management Plan
DBP: Diastolic blood pressure
ECG: Electrocardiogram
FACT-G: Functional Assessment of Cancer Therapy-General
FACT-B: Functional Assessment Cancer Therapy Breast
FACT-P: Functional Assessment Cancer Therapy Prostate
FAIR: Findable-Accessible-Interoperable-Reusable-principles of data sharing and management
FAPERGS: Fundação de Amparo à Pesquisa do Estado do Rio Grande do Sul (Research Support Foundation of the State of Rio Grande do Sul)
FIPE: Fundo de Incentivo à Pesquisa
GEE: General estimating equations
HCPA: Hospital de Clínicas de Porto Alegre-City Hospital at Porto Alegre
IPAQ: International Physical Activity Questionnaire
IRB: Institutional Review Board
ITT: Intention-to-treat analysis
LOCF: Last observation carried forward
OSF: Open Science Framework
PA: Physical activity
RCT: Randomized controlled trial
SBP: Systolic blood pressure
SPIRIT: Standard Protocol Items: Recommendations for Interventional Trials
WHO: World Health Organization
